# Does Pet Touch Influence Mental Health?

**DOI:** 10.1093/geroni/igaf122.842

**Published:** 2025-12-31

**Authors:** Taylor Pope, Patricia Thomas

**Affiliations:** Purdue University, West Lafayette, Indiana, United States; Purdue University, West Lafayette, Indiana, United States

## Abstract

Older adults are often at risk of social isolation and mental health consequences. Touch between humans has established benefits in reducing feelings of anxiety, depression, and loneliness; however, touch between humans and their pets may also improve the mental health of older adults. Applying stress process theory, we examine the relationship between pet touch and self-reported mental health using data from the National Social Life, Health, and Aging Project (NSHAP), a nationally representative longitudinal, population-based household survey of older adults in the United States. Our sample includes 1,815 participants aged 57 to 85 (Mean age = 68) across two rounds of data collection (2006-2011). Using a lagged dependent variable model, we assess whether pet touch in Round 1 predicts changes in mental health by Round 2, while controlling for socio-demographic and health-related factors. Greater pet touch in Round 1 was associated with improved mental health in Round 2 (B = .022, 95% CI: .003, .041, p = .021). Next, we examined an interaction between frequency of pet touch and marital satisfaction among the married/partnered, to understand whether pet touch was more important for the mental health of those experiencing low marital satisfaction or whether these two resources combined for particularly better mental health. Pet touch was not beneficial for those with low marital satisfaction. However, individuals with higher marital satisfaction and frequent pet touch experienced better mental health in Round 2. Our findings suggest that pet touch could benefit mental health, improving the wellbeing of older adults.

